# Psychological and Social Components of Recovery Following Anterior Cruciate Ligament Reconstruction in Young Athletes: A Narrative Review

**DOI:** 10.3390/ijerph18179267

**Published:** 2021-09-02

**Authors:** Emil Stefan Vutescu, Sebastian Orman, Edgar Garcia-Lopez, Justin Lau, Andrew Gage, Aristides I. Cruz

**Affiliations:** 1 Department of Orthopaedics, The Warren Alpert Medical School of Brown University and Rhode Island Hospital, Providence, RI 02903, USA; sebastian_orman@brown.edu (S.O.); edgar_garcia@brown.edu (E.G.-L.); ahgage13@gmail.com (A.G.); aristides.cruz@gmail.com (A.I.C.J.); 2 Boston College, Boston, MA 02467, USA; laujm@bc.edu

**Keywords:** ACL injury, ACL reconstruction, psychological readiness, return to play, knee

## Abstract

Anterior cruciate ligament (ACL) rupture is a common injury in young athletes. To restore knee stability and function, patients often undergo ACL reconstruction (ACLR). Historically, there has been a focus in this population on the epidemiology of ACL injury, the technical aspects of ACL reconstruction, and post-operative functional outcomes. Although increasingly recognized as an important aspect in recovery, there remains limited literature examining the psychological aspects of post-operative rehabilitation and return to play following youth ACL reconstruction. Despite technical surgical successes and well-designed rehabilitation programs, many athletes never reach their preinjury athletic performance level and some may never return to their primary sport. This suggests that other factors may influence recovery, and indeed this has been documented in the adult literature. In addition to restoration of functional strength and stability, psychological and social factors play an important role in the recovery and overall outcome of ACL injuries in the pediatric population. Factors such as psychological readiness to return-to-play (RTP), motivation, mood disturbance, locus of control, recovery expectations, fear of reinjury, and self-esteem are correlated to the RTP potential of the young athlete. A better understanding of these concepts may help to maximize young patients’ outcomes after ACL reconstruction. The purpose of this article is to perform a narrative review of the current literature addressing psychosocial factors associated with recovery after ACL injury and subsequent reconstruction in young athletes. Our goal is to provide a resource for clinicians treating youth ACL injuries to help identify patients with maladaptive psychological responses after injury and encourage a multidisciplinary approach when treating young athletes with an ACL rupture.

##  1. Introduction

Over 38 million children participate in organized sports in the United States [1]. In this cohort, anterior cruciate ligament (ACL) injuries are common in sports that require cutting, pivoting, and jumping. Female soccer players and male football players demonstrate the highest rate of ACL injuries, with an incidence of approximately 14 per 100,000 [1]. Other countries show a relatively similar incidence, as Scandinavian registries report 34 per 100,000 in Norway, 38 per 100,000 in Denmark, and 32 per 100,000 people in Sweden [2]. Germany reports 32 per 100,000 and New Zealand 37 per 100,000 people in two studies that use insurance claims for ACL reconstructions [2,3].

In order to mitigate loss of stability and function after an ACL rupture, some patients elect to undergo ACL reconstruction (ACLR) with subsequent post-operative rehabilitation. The postoperative success rate 3 years following ACLR is as high as 96%–98% [4]. In a 10+ year longitudinal study, Shelbourne and Gray found that 90% of patients who underwent ACLR exhibit normal International Knee Documentation Committee (IKDC) knee survey scores for range of motion, long term stability, and strength [5]. Additionally, over 85% of athletes achieved clinically satisfactory outcomes in terms of knee laxity, muscle strength, and single-leg hop distance [6]. However, despite successful ACLR technique and successful functional outcomes, a discrepancy still exists between recovered patients and the return to sport (RTS) rate at or above their preinjury level of sport [7,8].

Many athletes typically return to their sport 12 months following ACLR; however, as many as two-thirds do not regain their preinjury level of sport within this time frame [9]. Common reasons proposed for the lack of RTS include psychological readiness, fear of sustaining a new injury, and the reduced desire to return to sport [7,8,9,10,11,12,13]. These factors are modifiable and of interest to both researchers and physicians. 

This narrative review aims to compile and provide a comprehensive overview of the current understanding of the psychological factors associated with the recovery of youth athletes following ACLR. In March 2021, we searched the PUBMED and Google Scholar databases for literature evaluating psychological factors involved in ACL reconstruction recovery in young athletes, regardless of publication type or study type. Non-English studies were excluded from our review.

## 2. Psychological Factors Defined

Psychosocial factors have been shown to affect the mental wellbeing of individuals and their ability to recover from injury [14,15,16]. Increased levels of stress and decreased social support are highly correlated with a decrease in both physical and mental health [17]. These factors have also been observed to serve as post-operative predictors for pain and negative patient reported outcomes [18,19]. The specific psychological factors that affect patients’ RTS after ACLR include, but are not limited to. psychological readiness, fear of reinjury, and desire to return to sport.

Psychological readiness serves as a one of many predictors for an athlete’s ability to RTS following surgical intervention. Psychological readiness is characterized by low levels of impediments including fear and anxiety and higher levels of dsuch as confidence, motivation, and realistic expectations [20,21]. Psychological readiness is measured using the ACL-Return to Sport After Injury (ACL-RSI) and the Injury-Psychological Readiness Return to Sport (I-PRRS) scales [11,12,20]. The ACL-RSI is a patient reported questionnaire that utilizes concepts of emotions, confidence, and risk appraisal to provide specific measurements of psychological readiness to RTS or pre-injury level of sport [11,12,22]. The I-PRRS scale tracks a patient’s level of confidence at specific points during their rehabilitation [18], which is used to determine the patient’s psychological readiness to RTS [23]. The I-PRRS scale allows for the evaluation of psychological readiness irrespective of the type of injury, while the ACL-RSI scale is used solely to assess psychological readiness of individuals recovering from ACLR [20,22,23].

Fear of reinjury takes on the form of kinesiophobia [24]. Kinesiophobia is a construct within the fear-avoidance model that is defined as a state where an individual experiences excessive, irrational, and debilitating fear of physical movement and activity as a result of a feeling of susceptible to painful injury or reinjury [6,25,26]. The Tampa Scale of Kinesiophobia (TSK-11) is used to measure levels of pain-related fear of activity or re-injury in patients recovering from ACLR or other forms of surgical intervention, with higher scores being indicative of higher levels of fear [27,28,29].

Desire to return to sport or motivation stems from obtaining external/internal rewards and maintaining a positive self-concept [30]. Motivation holds a basis in autonomy or actions based on one’s interests and values and these interests compel individuals to comply with what will allow them to best achieve these interests and goals [31]. The assessment of motivation or the desire to RTS usually takes the form of interviews utilizing open-ended questions that provide researchers with a sense of a patient’s thoughts and experiences relating to their injury and rehabilitation [32].

### 2.1. Psychological Readiness

One of the predictors of successful return to play of the athlete and regaining a pre-injury level of functioning is the display of psychological readiness [9,11,12,33]. In other words, the athlete should develop a more positive mindset regarding his or her injury, and regarding the idea of returning to play. Using the ACL-RSI scale to evaluate psychological readiness, Ardern et al. found that a one point increase in ACL-RSI scale score (higher psychological readiness) equates to approximately twice the odds of returning to the preinjury activity [11]. Utilizing the same ACL-RSI scale, Kitaguchi et al. found that the RTS of competitive athletes 6 months following ACLR was most significantly predicted by ACL-RSI scores above 55 points, indicating a high psychological readiness [12]. Athletes who RTS exhibited significantly higher ACL-RSI scores (higher psychological readiness) than those who did not return [12].

Gender also appears to be related to psychological readiness to RTS in patients recovering from ACLR [34,35]. Utilizing the ACL-RSI scale to assess psychological readiness Webster et al. found that males reported higher levels of psychological readiness than females, with 30% of male athletes returning to preinjury level of sport as compared to 17% of their female counterparts. From the ACL-RSI scale, female patients were seen to score lower on psychological readiness and exhibit an increased negative outlook on their injury [34].

### 2.2. Fear of Reinjury

As many as 24%–35% of athletes who injure their ACL can suffer from subsequent fear of reinjuring, hindering their return to sport or to their preinjury level of sport [8,11,12,28,29,36]. Through patient interviews, Ross et al. found long post-operative recovery time and restricted function to be associated with the formation of fear of reinjury [37]. Based on the TSK-11 scale, athletes recovering from ACLR exhibited higher levels of fear (higher TSK-11 scores) and were less likely to RTS. However, patients who did end up returning to sport who reported higher levels of fear were seen to be at higher risk of reinjury [11]. Tagesson and Kvist found that patients who self-reported greater levels of fear of reinjury tended to suffer from an ACL graft rupture or a contralateral ACL rupture following RTS at higher levels than those athletes with lower fear of reinjury [38]. When combined with functional limitations, individuals exhibiting fear of reinjury are less likely to RTS due to mistrust of the knee, resulting from a noticeable lack of proper knee function and strength following ACLR [26]. Patients who reported a fear of reinjury as a barrier to their return to sport were more susceptible to longer term functional deficits 1 year post-ACLR [26]. More research is necessary to determine the extent to which fear of reinjury coincides with the increased risk of reinjury seen in patients returning to sport following ACLR.

### 2.3. Desire to Return to Sport

Athletes who desire to return to sport appear to be more likely to do so. Competitive athletes returned to sport within 12 months significantly more than recreational athletes, due to perceived increased time investment in the sport, leading to increased motivation to return [9]. Fältström et al. found that a single point increase in motivation (1 being low motivation and 10 being high motivation) equated to a 1.5-fold higher probability that an athlete would return to soccer following ACLR [36]. Those who exhibit higher levels of motivation are seen to comply with and complete their rehabilitation program, allowing for safer and quicker RTS and to preinjury level of sport [9,32,36].

The type of motivation to RTS varies by age. Brewer et al. found that older participants (30–40 years) who relied on self-motivation and social support had higher rates of completion of home rehabilitation exercise regimens for ACLR [39], whereas younger patients tended to rely on self-identification as an athlete, rather than the self-motivation seen in older patients [39]. The inability to RTS for younger athletes hinders athletic identity and social support, resulting in a negative association with both knee laxity, home exercise completion, and home cryotherapy completion [12,38,40].

Hildingsson et al. found that motivational sources can include personal goals in the sport, passion for the sport, a strong athletic identity, and the social aspect of being on a sports team [32]. The social environment surrounding the athlete has been seen to play an important role in their levels of motivation due to the inherent ability to stimulate a sense of autonomy and contribute to an increased feeling of competence, independence, and relatedness [32,41], allowing them to better rehabilitate their injury and RTS.

Patients’ desire to return to sport is also tied to the amount of time they have invested in the sport, with higher levels of motivation to complete rehabilitation protocols demonstrated in the more devoted, competitive athletes [39]. Ardern et al. found that elite athletes with high levels of investment in their sport had twice the odds of returning to their preinjury level of play and six times the odds of returning to competitive sport as non-elite athletes. The authors speculated that these findings are tied to the perceived financial benefit that may ensue from return to play [7].

## 3. Reinjury

Psychological factors have been linked to reinjury after successful return to sport. One-third of young athletes who undergo primary ACLR will rupture their graft or injure the contralateral ACL within two years following ACLR [42]. The incidence of a second ACL injury in young athletes (mean age, 17.2 ± 2.6 years) within 24 months of ACLR and RTS was six times greater than that of a young athlete with no history of ACL injury [43]. Interestingly, Webster et al. found that greater psychological readiness in younger patients correlated with a higher risk of a second ACL injury [44]. As such, the effects of these psychological factors do not simply end following RTS and continue playing a role in the likelihood of reinjury in those who RTS following ACLR. The Tampa Scale of Kinesiophobia (TSK-11) is a test that assesses pain-related fear of movement/reinjury. Individuals with TSK-11 scores higher than 19 at the point of RTS were 13 times more likely to suffer a second, ipsilateral ACL tear within 24 months following RTS [45]. Even following RTS, psychological and physical factors must be taken into account and steps must be taken in order to ensure that these factors do not allow subsequent ACL injuries to arise.

## 4. Treatment and Interventions

Proposed treatment focuses on postoperative psychological rehabilitation based on preoperative psychological assessment aimed at recognizing positive or negative psychological responses to injury. Evaluation of high versus low self-esteem, high versus low self-efficacy, optimism versus pessimism, and confidence versus anxiety can be a first step in treatments aimed at modifying harmful attitudes. Psychological rehabilitation is deemed necessary due to the inherent difference in timeline that exists between reaching physical readiness versus psychological readiness [20,46,47]. Psychological rehabilitation focuses on those who are more susceptible to the negative effects of the psychological factors of ACLR rehabilitation and RTS [20]. Hsu et al. hypothesized that addressing the psychological factors that have been previously identified such as fear of reinjury would assist patients in their RTS and preinjury level of sport [6]. Moving away from the fear of reinjury, interventions could also focus on addressing the desire or motivation to return to sport. Interestingly, since motivational factors such as perceived financial benefit from RTS appear to be positively correlated to RTS and return to preinjury level of sport [7], psychological rehabilitation that focuses on identifying the motivational factors to RTS or encourages the development of motivational factors could result in accelerated, successful RTS and to preinjury level of sport. Preoperative psychological assessments could be used to identify a lack of motivational factors prior to ACLR, which then allows doctors to attempt to help the patients pinpoint motivational factors that would assist the patient in their rehabilitation and successful RTS.

## 5. Summary & Recommendations

Through testing the psychological readiness in patients, it was shown that both fear of reinjury and motivation play a large role in the determination of psychological readiness [9,20,21,32,36,39].

With a high reinjury rate of 24%–35%, some patients recovering from ACLR exhibit fear of secondary injury [9,11,12,13]. Those exhibiting high levels of fear following ACLR are less likely to RTS compared to patients with lower fear scores as tested by TSK-11 [11,26,27,28,29]. However, the extent to which fear of reinjury is associated with the actual rate of reinjury has yet to be determined and further research is necessary.

Individuals who desire to return to sport are significantly more likely to RTS or to their preinjury level of sport [9,32,36,39]. Increased motivation correlates with increased home exercise completion [9,32,36,39,40]. While self-motivation plays a role in the RTS in older individuals, athletic self-identification serves as a form of motivation in younger athletes [39]. Additionally, patients with more time invested into their sport prior to injury exhibit an increased likelihood of RTS—almost double the likelihood in elite athletes [7]—and preinjury level of sport [11]. It seems that the time invested in a sport and increased motivation to RTS correlate to the perceived financial benefit that comes from RTS; therefore further research is needed to determine whether the development and identification of strong motivational factors would increase the likelihood of RTS and preinjury level of sport in athletes who appear to lack specific motivational factors. 

While increased psychological readiness appears to be positively associated with RTS, such increases also appear to correlate with an increased risk of reinjury of as high as six times [43] following RTS and return to preinjury level of sport. As such, those who have RTS and to a preinjury level of sport must continue to be monitored due to the lasting effects of psychological factors that predict the increased occurrence of graft rupture and contralateral ACL tear [38].

The current recommended interventions include psychological rehabilitation in addition to physical rehabilitation to facilitate successful RTS and return to preinjury level of sport [6,20,46,47]. Psychological rehabilitation addresses the psychological factors that have been previously discussed (psychological readiness, fear of reinjury, and desire to return) to assist patients in their successful RTS. Two randomized control trials from New Zealand examine the effectiveness of a modeling video and imagery, respectively to reduce preoperative perceptions of anxiety and pain as well as improve functional outcomes [48,49]. In both studies the study group experienced less anxiety and knee function as measured by the IKDC system and knee laxity. Current literature lacks detailed experimentation surrounding the specific outcomes of psychological rehabilitation; however, current research documents their hypothesized benefits in allowing for safe, successful RTS.

The conclusions drawn from this review will help provide recommendations for medical professionals treating young patients recovering from an ACL injury whose recovery may be impeded by psychological challenges. Ultimately, this may facilitate future research regarding postoperative psychological rehabilitation after ACLR.

A limitation of this study is that the design was not a systematic review and not all relevant literature was considered for inclusion. The quality of the the articles’ content was not critically evaluated and selection of the articles was that solely of the authors, therefore there was potential for selection and interpretation bias.

## 6. Conclusions

This review summarized the psychological factors that can influence ACLR rehabilitation of athletes hoping to RTS or return to preinjury level of sport. We identified and defined the psychological factors, psychological readiness, fear of reinjury, and desire to return, all of which have been demonstrated to correlate with RTS and return to preinjury level of sport for patients recovering from ACLR. Related literature indicates that individuals who exhibit higher levels of psychological readiness are more likely to RTS and return to preinjury level of sports.

Future research surrounding psychological rehabilitation and care both pre- and post-operatively is necessary to better understand how patients can more successfully RTS without the consequences that result from negative psychological factors such as high levels of fear and decreased levels of motivation.

## Data Availability

Not applicable.
